# Case Report: Rare duodenal schwannoma diagnosis and treatment process report

**DOI:** 10.3389/fonc.2025.1528653

**Published:** 2025-03-06

**Authors:** Shan Li, Lingyu Tu, Ting Li, Xiongchuan Pei, Xijin Wang, Yanqing Shi

**Affiliations:** ^1^ Jiangxi Provincial Key Laboratory of Cell Precision Therapy, School of Basic Medical Sciences, Jiujiang University, Jiujiang, China; ^2^ Department of Pathology, Affiliated Hospital of Jiujiang University, Jiujiang, China; ^3^ Department of Gastroenterology, Affiliated Hospital of Jiujiang University, Jiujiang, China

**Keywords:** endoscopic submucosal dissection, duodenal schwannoma, case report, S-100, immunohistochemical (IHC)

## Abstract

**Background:**

Duodenal schwannoma is a rare benign mesenchymal tumor originating from the Schwann cells of peripheral nerves. Its accurate diagnosis remains challenging owing to non-specific clinical and radiological features.

**Case presentation:**

This report describes the clinical diagnosis and treatment of a duodenal schwannoma in a 30-year-old female patient. An initial outpatient gastroscopy revealed a submucosal lesion in the duodenum, with differential diagnoses including ectopic pancreas or gastrointestinal stromal tumor. Furthermore, plain and contrast-enhanced abdominal CT scans showed a nodule protruding into the duodenal bulb, suggesting a benign lesion. The patient underwent endoscopic submucosal dissection to remove the tumor. During the procedure, a 1.0 × 1.6 cm submucosal nodule was identified on the anterior wall of the duodenal bulb, with a mildly eroded and slightly depressed surface. The lesion was firm and non-mobile. Histopathological examination of the resected specimen confirmed a spindle cell tumor originating from the duodenal mucosa, leading to a definitive diagnosis of duodenal schwannoma.

**Conclusion:**

Duodenal schwannoma is a rare submucosal tumor traditionally treated with surgical resection. This case highlights the safety and efficacy of endoscopic submucosal dissection in the treatment of duodenal schwannomas, allowing complete tumor resection while preserving gastrointestinal function. In addition, early detection and complete resection are key for preventing recurrence and potential complications.

## 1 Introduction

Schwannomas are benign tumors that originate from Schwann cells that form the myelin sheath of peripheral nerves (1). Although these tumors can occur in any part of the body, gastrointestinal schwannomas are extremely rare, accounting for less than 3% of all gastrointestinal stromal tumors (GISTs) (2). Among gastrointestinal schwannomas, the duodenum is a particularly rare site, with most cases occurring in the stomach or colon (3–5).

Duodenal schwannomas typically grow slowly and are often asymptomatic, which may lead to their incidental discovery during imaging or endoscopic examinations for unrelated diseases. Symptoms are usually non-specific and may include abdominal pain, nausea, vomiting, or gastrointestinal bleeding. Owing to the non-specificity of these symptoms, duodenal schwannomas are prone to be misdiagnosed as other types of submucosal tumors, such as GISTs, adenocarcinoma, or neuroendocrine tumors (6).

Histopathological examination is crucial for a definitive diagnosis of schwannomas. The tumors are characterized by spindle-shaped cells arranged in the Antoni A and B regions and positive immunohistochemical staining of the S-100 protein, confirming their origin from Schwann cells. Unlike GISTs, schwannomas typically do not express c-KIT (CD117) or DOG1, which aids in their differentiation from other submucosal tumors (7, 8).

Currently, the preferred treatment for duodenal schwannomas is surgical resection, which can be accomplished via open surgery or endoscopic techniques, depending on the size and location of the tumor (9, 10). Given its benign nature, complete resection generally leads to a favorable prognosis, with a low risk of recurrence or malignant transformation. Nevertheless, close follow-up is recommended to monitor for recurrence or complications. Backes et al. assert that endoscopic submucosal dissection (ESD) can achieve complete resection of lesions, reduce the risk of recurrence, and is comparable in efficacy to surgical procedures, while featuring less trauma, lower cost, and a shorter hospital stay (11). This article reports a rare case of duodenal schwannoma and elaborates on its clinical manifestations, diagnostic difficulties, and treatment methods to supplement the lack of relevant literature on duodenal schwannomas.

## 2 Case description

### 2.1 General information

A 30-year-old female patient was admitted to the hospital after a duodenal tumor was incidentally discovered during a routine health check-up, with the tumor present for approximately 2 weeks. The patient had an unremarkable medical history, except for a cesarean section. She had no history of blood transfusions and denied any known drug or food allergies.

On admission, the patient’s vital signs were as follows: temperature, 36.5°C; pulse, 65 beats/min; respiratory rate, 18 breaths/min; and blood pressure, 110/76 mmHg. The patient was alert and well-oriented. Cardiopulmonary auscultation revealed no significant abnormality. The abdomen was soft, with no tenderness, rebound tenderness, or guarding. Murphy’s sign was negative and McBurney’s point was non-tender. The liver and spleen were not palpable below the costal margin, and there was no tenderness on percussion in the liver region. No tenderness was noted in either flank. The shifting dullness was negative, and bowel sounds were heard at a frequency of five times per min. Edema was not observed in the lower extremities.

The initial clinical assessment indicated no obvious abnormalities, and the patient was scheduled for further evaluation and treatment for the duodenal tumor.

### 2.2 Diagnosis and treatment

**Figure 1:**
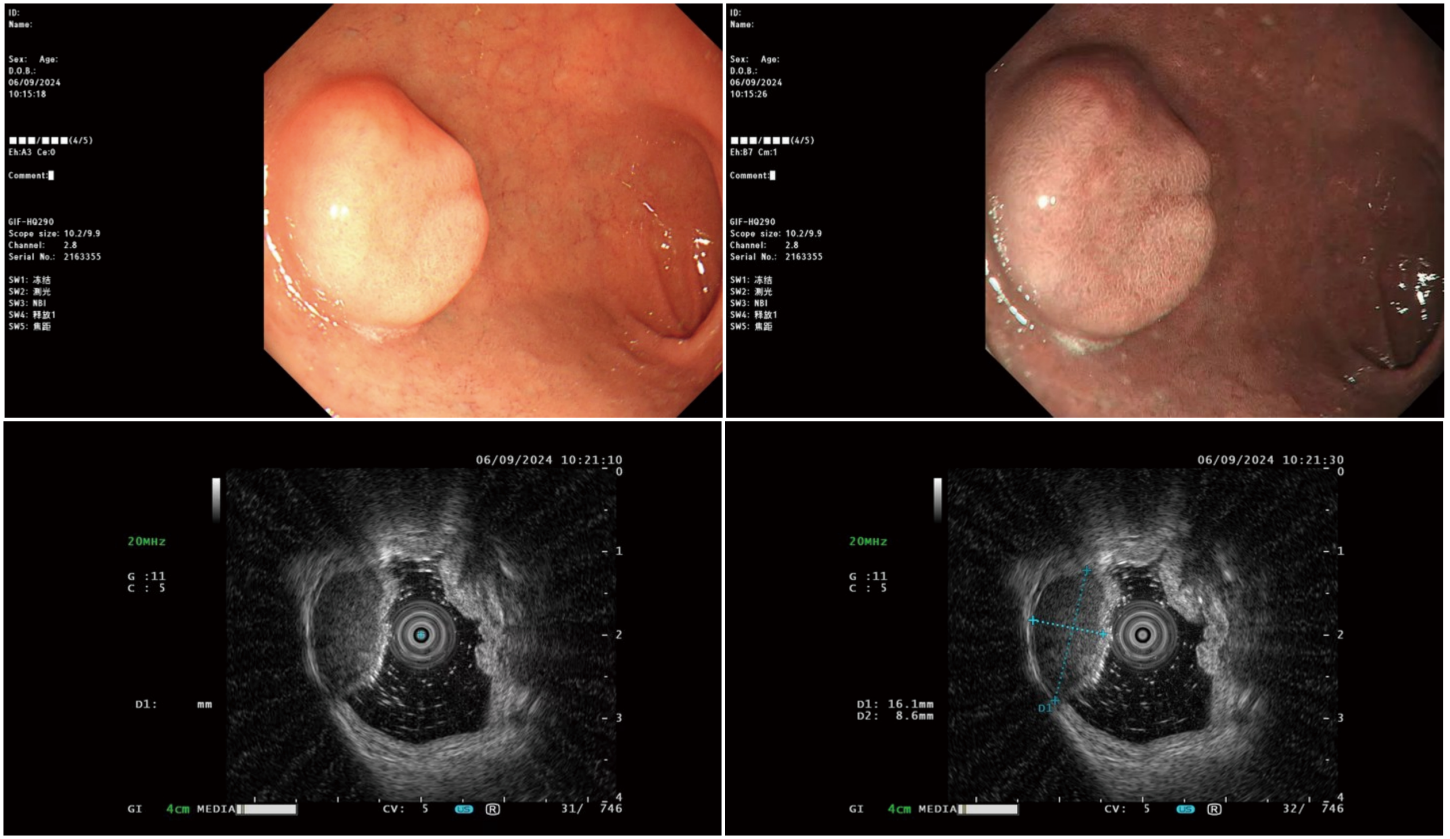
Ultrasound colonoscopy examination.

Pre-admission outpatient gastroscopy: On September 6, 2024, ultrasonic gastroscopy performed at the Affiliated Hospital of Jiujiang University suggested submucosal protrusion of the duodenum (ectopic pancreas? Stromal tumor)? (**Figure 1**). On September 12, 2024, further diagnostic evaluations were performed: routine blood tests were normal; blood biochemistry indicated that liver function, renal function, and serum creatinine, serum uric acid, serum electrolyte, serum glucose, and blood lipid levels were all normal; hepatitis B serological markers and coagulation function were normal; and serum digestive system tumor markers, such as alpha-fetoprotein, carcinoembryonic antigen, carbohydrate antigen CA19-9, and carbohydrate antigen CA72-4, were all normal. Electrocardiogram results were within the normal range. The results indicated no significant abnormalities in laboratory or cardiac evaluations. These findings suggested that the duodenal lesion was unlikely to be associated with systemic diseases or malignancies.

**Figure 2:**
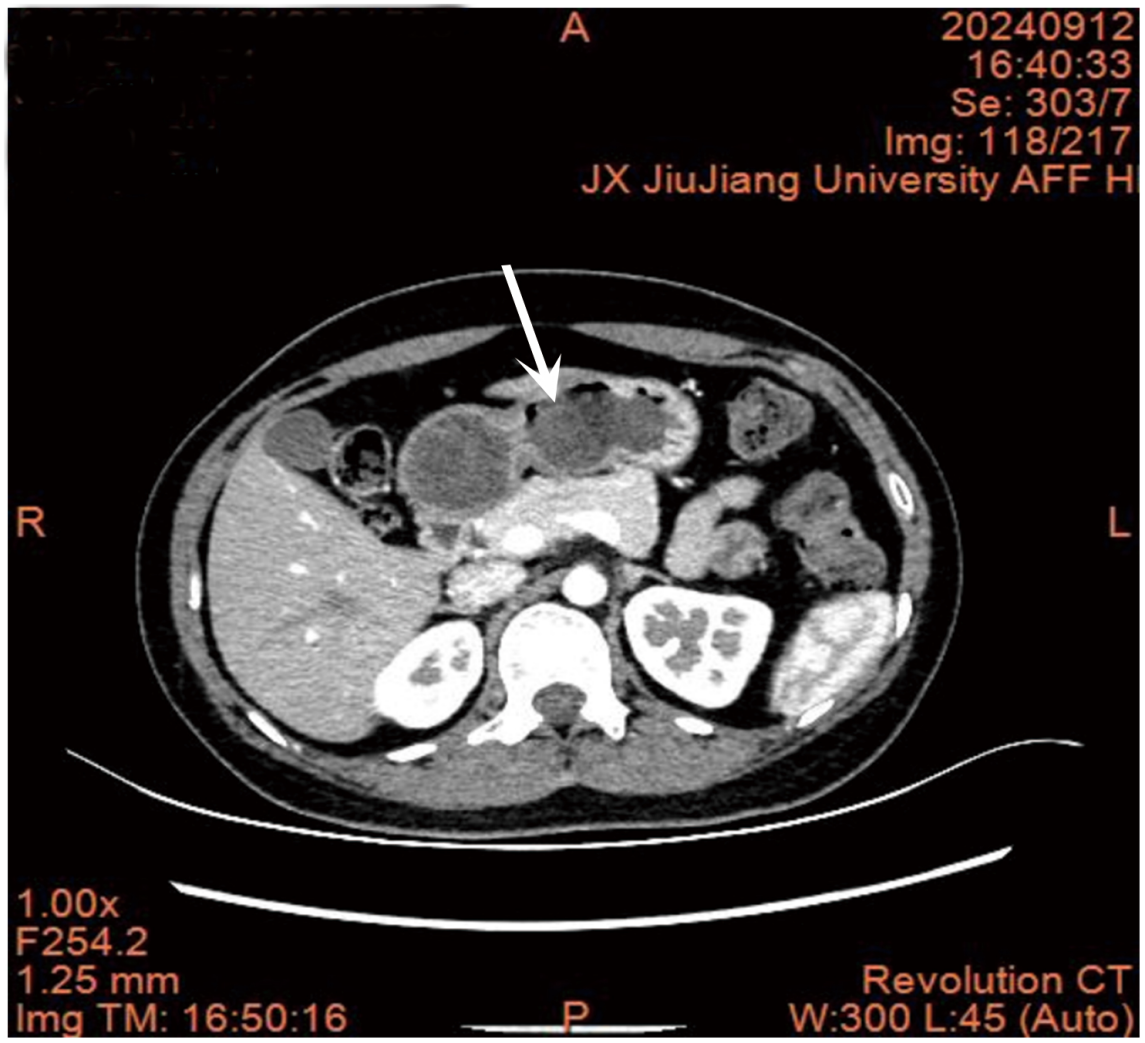
Abdominal CT scan results.

An upper abdominal plain scan and enhanced CT examination suggested the following. 1. A nodule protruding into the lumen of the duodenal bulb was considered benign with a high probability (**Figure 2**). 2. A small cyst in the left kidney. 3. Ground-glass nodules in the middle lobe of the right lung, and solid nodules in the lower lobe of the left lung and the upper lobe of the right lung, and annual follow-up was recommended. 4. Calcification in the lower lobe of the right lung.

**Figure 3:**
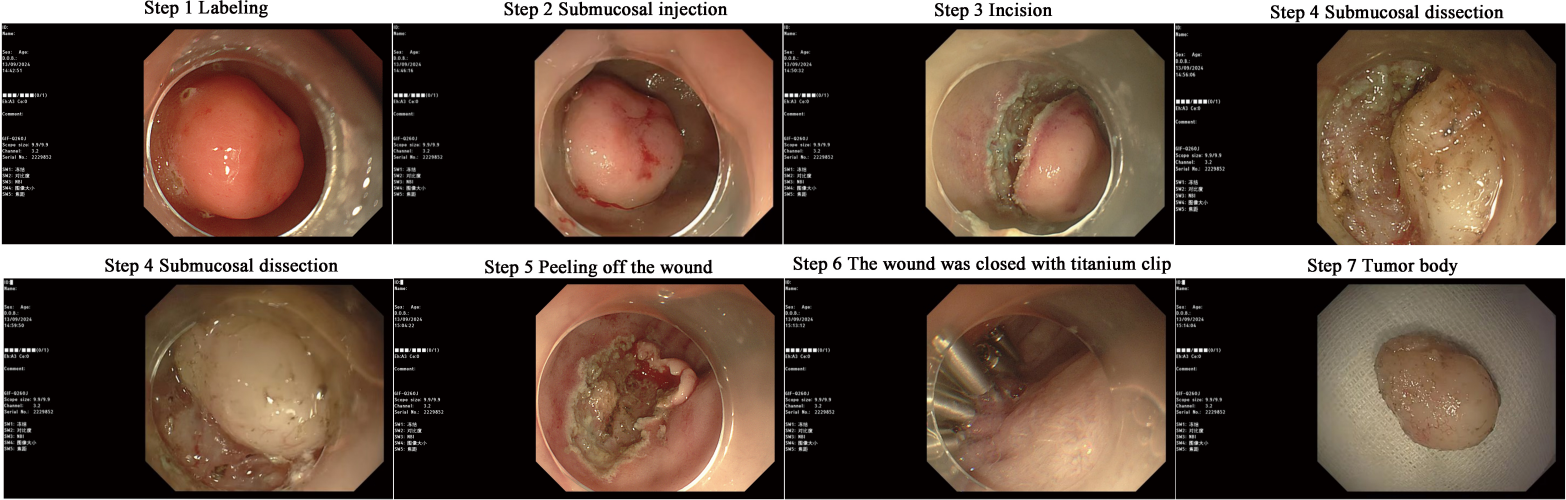
Endoscopic submucosal dissection surgery.

On September 13, 2024, the patient underwent ESD of a duodenal submucosal tumor under tracheal intubation anesthesia. During the procedure, a submucosal protrusion measuring approximately 1.0 × 1.6 cm was observed in the anterior wall of the duodenal bulb. The lesion exhibited mild erosion and slight depression, with a firm texture and no mobility. Circumferential markings were made around the lesion by using a disposable mucosal incision knife (Victor Med). Submucosal injections were administered using a syringe with saline solution mixed with 1:10,000 adrenaline. A circumferential incision was made through the superficial mucosa using a cutting knife, followed by gradual dissection of the submucosal layer. During dissection, the tumor was found to originate from the muscularis propria, and dissection was continued until the tumor was completely detached. A minor perforation occurred during the procedure, which was successfully closed using five titanium clips. No postoperative bleeding or perforation was observed. The entire surgical process was recorded (**Figure 3**), and a video of the procedure is shown in **Supplementary Video 1**. The initial diagnosis was a duodenal submucosal tumor, and an ectopic pancreatic or stromal tumor was also considered. The resected tissues were sent to the pathology department for examination.

**Figure 4:**
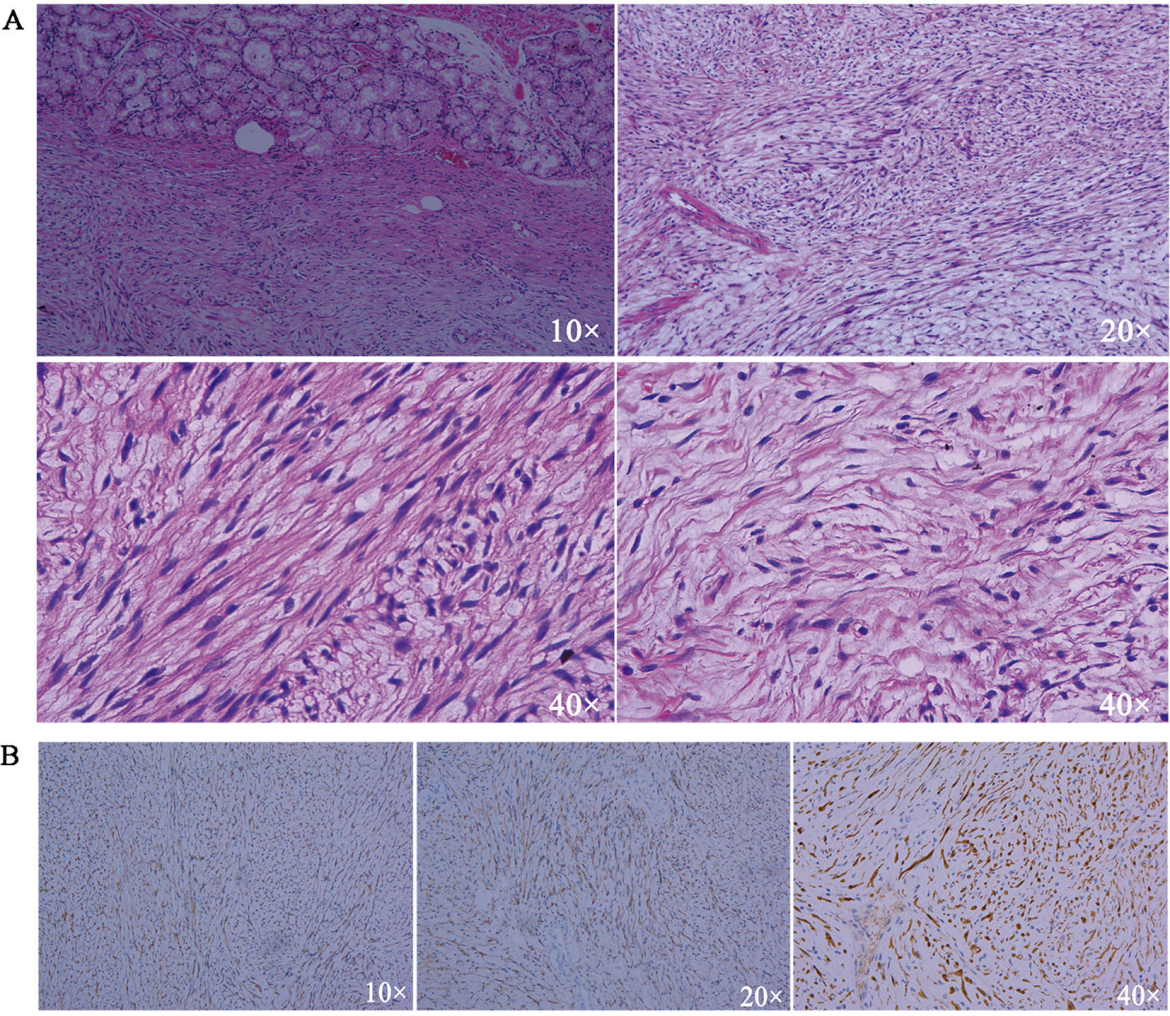
**(A)** HE staining results. **(B)** Immunohistochemical results of S-100.

Postoperative pathology confirmed the diagnosis of a spindle cell tumor of the duodenum, which was identified as a duodenal schwannoma (**Figure 4A**). Immunohistochemical staining results showed: SMA (–), Desmin (–), S-100 (+), Dog-1 (-), CD117 (-), CD34 (-), SDHB (+), and Ki-67 (approximately 1% +) (**Figure 4B**). These findings confirmed the diagnosis of a duodenal schwannoma.

Based on the aforementioned examination results, the patient was diagnosed with a duodenal schwannoma.

## 3 Discussion

Schwannoma is a benign tumor originating from the perineural sheath cells and myelin-forming cells of the peripheral nervous system and can occur in any tissue or organ containing nerve fibers. It typically occurs in individuals aged between 20 and 50 years, with a peak incidence in women aged approximately 50 years. It is most commonly found in the head and face, extremities, and central nervous system but can also occur in the retroperitoneum and gastrointestinal tract. Schwannomas arising from the Auerbach’s plexus of the muscularis mucosae of the gastrointestinal tract are relatively rare, accounting for only 2.9% of gastrointestinal interstitial tumors. It was believed that the incidence of duodenal lesions is low. However, with the development of endoscopic technology and increased awareness of duodenal lesions among clinicians, the detection rate has increased from 0.3% to 4.6% (12–14). Clinically, the most common site of occurrence is the gastrointestinal tract, accounting for 60% to 70%, followed by the colon and rectum, with less common occurrences in the small intestine and esophagus, especially in duodenal schwannomas, which are often reported in case form in the literature (15).

With advancements in diagnostic techniques, such as high-resolution endoscopy and abdominal imaging (CT and MRI), clinicians are more likely to detect submucosal tumors in the duodenum that might have gone unnoticed in the past (16). Increased awareness of gastrointestinal lesions among clinicians has also contributed to this enhanced detection. As demonstrated in this case, duodenal schwannomas may be discovered incidentally during routine health check-ups or endoscopic procedures performed for unrelated reasons. The benign nature of these tumors often makes them asymptomatic until they are discovered by imaging or endoscopy. We emphasize the importance of considering duodenal schwannomas in the differential diagnosis of other gastrointestinal submucosal tumors, such as GISTs, ectopic pancreas, and neuroendocrine tumors, as these conditions may present with similar clinical and radiological features. We highlight how endoscopic procedures, including gastroscopy and endoscopic ultrasound, are increasingly used to identify and evaluate submucosal lesions, providing a more accurate assessment of the duodenal wall structure and enabling early detection of rare lesions such as schwannomas.

Duodenal schwannomas are rare submucosal tumors that present diagnostic and therapeutic challenges because of their non-specific clinical and imaging features. Traditional treatment approaches, such as surgical resection (e.g., local excision or pancreaticoduodenectomy), are the standard treatments for managing duodenal tumors (17). However, these methods often involve major trauma, increase the risk of complications, prolong the recovery time, and reduce the quality of life of patients, especially when dealing with benign lesions such as schwannomas (18, 19). In contrast, ESD is a minimally invasive procedure with several unique advantages (20). ESD can be performed en bloc to ensure complete resection of the tumor and preservation of gastrointestinal function. This technique reduces patient morbidity, shortens the recovery time, and avoids the need for invasive surgical procedures (21, 22). Compared with pancreaticoduodenectomy, which is traditimplex or malignant lesions, ESD is a safer and less invasive option for benign tumors such as schwannomas. Surgical procedures have higher morbidity rates and longer hospital stays, whereas ESD minimizes surgical trauma and preserves the integrity of surrounding tissues. We demonstrated that ESD yielded comparable or better results than surgery, particularly in terms of resection success and post-treatment quality of life. This case highlights the efficacy and safety of ESD as an alternative to surgery for treating duodenal schwannomas. Long-term follow-up is necessary to monitor for recurrence, and ESD holds promise for broader application in managing other rare gastrointestinal submucosal tumors. Future studies are essential to further refine ESD protocols and evaluate its long-term outcomes, enabling its integration as a standard approach for the treatment of rare duodenal lesions.

In summary, duodenal lesions are relatively less common than lesions in other parts of the gastrointestinal tract, but their detection rates are increasing. The diagnosis of a duodenal schwannoma is difficult before surgery and is usually confirmed by pathological examination during or after surgery. ESD can achieve large-scale and curative resection, and has been well proven in the endoscopic treatment of esophageal, gastric, and colonic tumors, with satisfactory safety (23–25). By improving the endoscope and cutting device types, as well as postoperative closure techniques for mucosal defects, the incidence of complications can be effectively reduced (26). However, continuous experience summarization and improvements in duodenal ESD are needed to provide precise treatment to patients, reduce complications, and improve surgical satisfaction. We believe that ESD technology can be a good choice for minimally invasive treatment of duodenal lesions in the future.

## 4 Conclusions

Duodenal schwannomas are rare submucosal tumors that require accurate histopathological and immunohistochemical diagnosis. This case highlights ESD as a minimally invasive and effective treatment option that offers faster recovery, fewer complications, and preserves gastrointestinal function compared with traditional surgery. Despite their benign nature, regular follow-ups are essential to monitor for the recurrence of duodenal schwannomas. This case underscores the potential of ESD in managing rare gastrointestinal lesions and calls for further studies to confirm its long-term efficacy.

## Data Availability

The original contributions presented in the study are included in the article/**Supplementary Material**. Further inquiries can be directed to the corresponding author.

## Supplementary material

SUPPLEMENTARY VIDEO 1 Surgical video.
